# Comparative genomic hybridization and chromosomal instability in solid tumours

**DOI:** 10.1038/sj.bjc.6690433

**Published:** 1999-05

**Authors:** P H Rooney, G I Murray, D A J Stevenson, N E Haites, J Cassidy, H L McLeod

**Affiliations:** Departments of Medicine and Therapeutics, Pathology and Medical Genetics, University of Aberdeen, Foresterhill, Aberdeen, AB25 2ZD, UK

**Keywords:** comparative genomic hybridization, solid tumour genetics, chromosomal instability


*British Journal of Cancer* (1999) **80**(5/6), 862–873
© 1999 Cancer Research Campaign
Article no. bjoc.1998.0433

### Comparative genomic hybridization and chromosomal instability in solid tumours

**PH Rooney[1], GI Murray[2], DAJ Stevenson[3], NE Haites[1,3], J Cassidy[1] and HL McLeod[1]**

Departments of [1]Medicine and Therapeutics, [2]Pathology and [3]Medical Genetics, University of Aberdeen, Foresterhill, Aberdeen AB25 2ZD, UK

**Keywords**: comparative genomic hybridization; solid tumour genetics; chromosomal instability

The rational development of new diagnostic or prognostic tumour markers and the identification of novel cellular targets for anti-cancer chemotherapy relies on a more definitive understanding of tumour biology. Classical approaches using cellular pharmacology, and more recently molecular pharmacology, have led to the discovery of a number of growth factors and their receptors as well as other proteins which has resulted in novel therapies (e.g. inhibitors of epidermal growth factor receptor tyrosine kinase) and prognostic markers (e.g. oestrogen receptor levels in breast cancer) (Levitzki et al, 1995; Dowsett et al, 1997). Using classical metaphase cytogenetic techniques, many chromosomal aberrations have been identified in human cancer cell lines and primary culture of haematological malignancies. This chromosomal information has facilitated identification of a number of impor– tant genes associated with tumorigenesis (e.g. loss of chromosomal material on 13q led to identification of tumour suppressor gene RB1; Vogel, 1979). However, the use of metaphase cytogenetic analysis has been limited in solid tumours, mainly due to the difficulties in growing primary cultures in which to generate tumour metaphase chromosomes. However, this changed with the development of comparative genomic hybridization (CGH) and its ability to globally assess the genome of solid tumours for areas of loss and/or gain without the need for tissue culture (Kallioniemi et al, 1992; Forozan et al, 1997; Ried et al, 1997). CGH involves a competitive in situ hybridization of fluorescently labelled tumour DNA and healthy control DNA to normal metaphase chromosomes (Figure 1). Computer-assisted fluorescence microscopy is then used to assess the intensity of fluorochrome across each human chromosome. The differences in tumour and control fluorescence intensity along each chromosome on the reference metaphase spread are a reflection of the copy number changes of corresponding sequences in the tumour DNA. If chromosomes or chromosomal subregions are present in identical copy number within both the tumour and the normal DNA, an equal contribution from each fluorochrome is seen. However, a change in the fluorescent signal is seen if certain chromosomal subregions are gained or lost in the tumour DNA (Figure 1). The intensity of this signal is proportional to the amount of gain and loss seen for each region in the tumour DNA (Kallioniemi et al, 1992; Forozan et al, 1997). Regions with a high level of heterochromatin and centromeric regions are not informative with CGH. CGH data for the p regions of acrocentric chromosomes (e.g. 13p, 14p and 15p) must be interpreted with caution as repetitive sequences in these regions can affect the efficiency of competitive hybridization. With current technology, CGH has a theoretical limit of detection for gain and loss of genetic material of 5–10 Mb. However, gain of DNA in regions as small as 50 kb have been described in situations where high level amplification has occurred (Ried et al, 1997).

*Received 17 July 1998*
*Revised 28 September 1998*
*Accepted 21 October 1998*

*Correspondence to:* HL McLeod

Initial studies with CGH were restricted to DNA prepared from fresh or snap-frozen tumour material. More recently, technical advances have allowed the extraction of DNA from formalin-fixed paraffin-embedded sections through the use of degenerate oligonucleotide primed polymerase chain reaction (DOP-PCR) (Isola et al, 1994; Kuukasjarvi et al, 1997). The DOP-PCR technique allows genome-wide amplification of tumour DNA from nanogram quantities to the micrograms needed for CGH, and has enabled retrospective analysis of genomic loss and gain to be performed using DNA from archival material.

Although CGH analysis has been performed in a wide variety of adult and paediatric tumours, these results have not been extensively interpreted in the context of the CGH findings from other tumour types. In this review, the results of CGH analysis in 27 tumour types are evaluated to identify regions of loss or gain which are common to all malignancies as well as those which are specific for a given tumour type or tumour subtype. In addition, the degree of overall genomic instability for specific tumour types has been assessed.

### REVIEW OF THE LITERATURE

The Institute for Scientific Information (ISI) database from March 1992 to August 1998 identified 100 papers which described CGH findings in 2210 solid tumours of 27 cancer types (Appendix). This included common tumours (colon, breast, lung), gender-specific tumours (ovarian, cervix, testicular, prostate), paediatric tumours (neuroblastoma, rhabdomyosarcoma) and less common tumours (brain, renal, uveal melanoma). For each paper, the patterns of loss and gain in the p and q arms of each chromosome were recorded separately. Such an approach may not always be sufficient, as variation in subregions of the same chromosomal arm could be masked in some cases. However, a narrower definition for regions of gain and/or loss was not possible due to differences in the way CGH results have been presented in the

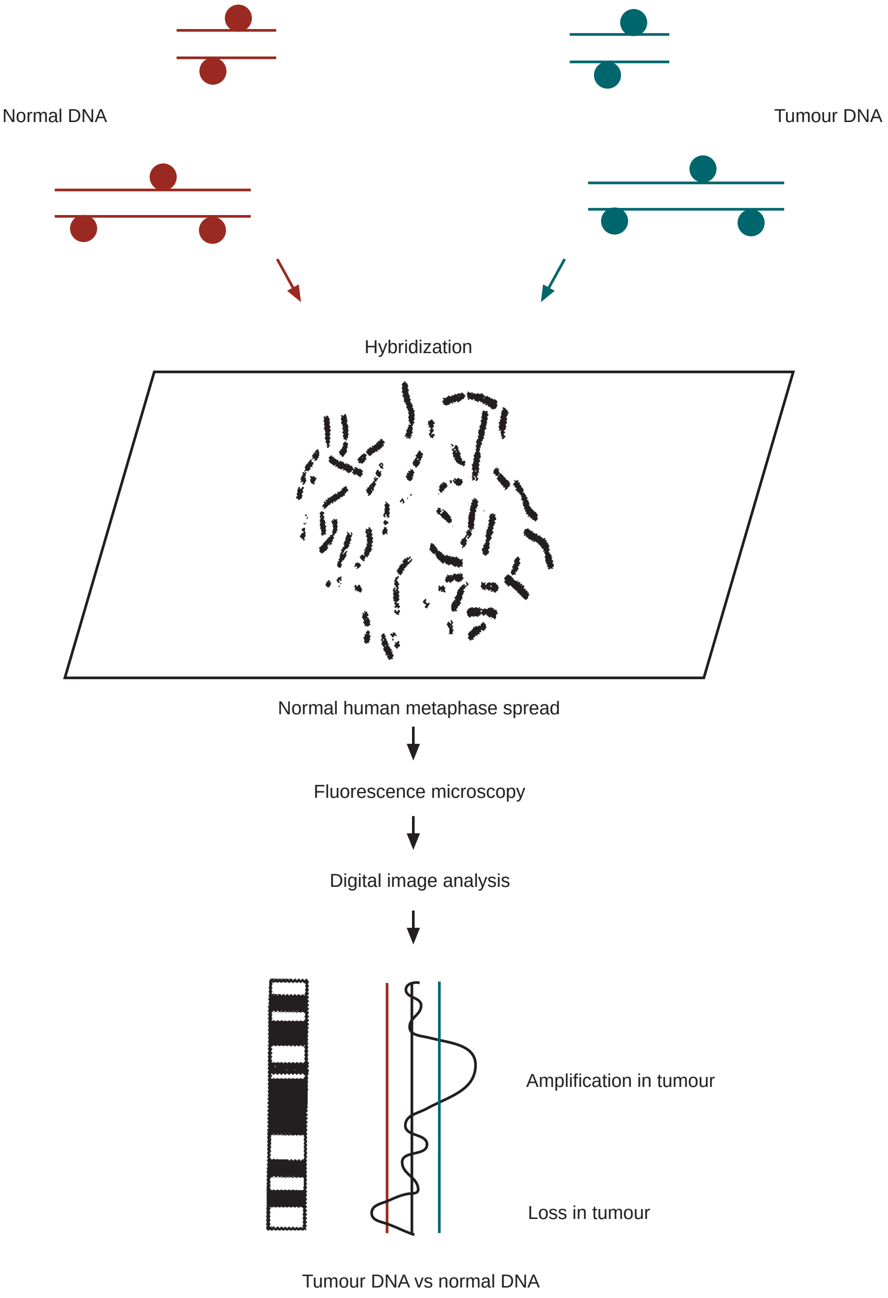

**Figure 1** A typical CGH experiment. Fluorescently labelled tumour DNA and reference DNA are competitively hybridized to donor human chromosomes. Using fluorescent microscopy the level of signal from the fluorescent DNA is assessed for each chromosome. For each chromosome a profile of the level of fluorescence is generated on CGH interpreting software. In most cases at least 10 chromosomes are assessed and an average of the fluorescence is generated. This allows regions of loss and gain that are consistently changed to be detected for a particular tumour sample literature. Studies of CGH in patients with leukaemia, lymphoma or studies with incomplete details of results for individual chromosomes were not included in this review. Cell line data were not included due to the difficulty in differentiating between initial chromosomal aberrations and those 'acquired' during cell culture. The frequency of overall loss or gain for each chromosome arm was determined by pooling the data from all tumours, from a given tumour type and from specific tumour subtypes.

### PATTERNS OF CHROMOSOMAL LOSS AND GAIN

### Solid tumours

The frequency of loss or gain for each chromosome arm was determined for all the solid tumours by pooling the data found in the literature for 2210 tumours (Table 1). Gain of chromosomal material was found more frequently than loss among the solid

© Cancer Research Campaign 1999

**Table 1** Loss and gain for each chromosomal arm when available CGH data from 2210 tumours (including 27 different solid tumour types) were pooled

| Chromosomal region | Total tumour *n* = 2210 | Gain (%) | Chromosomal region | Total tumour *n* = 2210 | Loss (%) |
|---|---|---|---|---|---|
| 8q + gains | 616 | 27.7 | 13q – losses | 363 | 16.3 |
| 1q + gains | 558 | 25.1 | 9p – losses | 357 | 16.1 |
| 7q + gains | 513 | 23.1 | 8p – losses | 333 | 15 |
| 7p + gains | 477 | 21.5 | 10q – losses | 304 | 13.7 |
| 17q + gains | 412 | 18.5 | 3p – losses | 297 | 13.4 |
| 3q + gains | 365 | 16.4 | 4q – losses | 297 | 13.4 |
| 20q + gains | 344 | 15.5 | 6q – losses | 296 | 13.3 |
| 5p + gains | 292 | 13.2 | 17p – losses | 260 | 11.7 |
| 12q + gains | 290 | 13.1 | 18q – losses | 245 | 11 |
| 12p + gains | 277 | 12.5 | 1p – losses | 226 | 10.2 |
| 11q + gains | 252 | 11.3 | 11q – losses | 218 | 9.8 |
| 6p + gains | 246 | 11.1 | 5q – losses | 206 | 9.1 |
| 20p + gains | 223 | 10 | 10p – losses | 202 | 9.1 |
| 19q + gains | 223 | 10 | 16q – losses | 196 | 8.8 |
| 2p + gains | 214 | 9.6 | 4p – losses | 188 | 8.5 |
| 13q + gains | 205 | 9.2 | 22q – losses | 184 | 8.3 |
| 19p + gains | 203 | 9.1 | 14q – losses | 183 | 8.2 |
| 1p + gains | 201 | 9 | 9q – losses | 170 | 7.7 |
| 14q + gains | 201 | 9 | 11p – losses | 167 | 7.5 |
| 2q + gains | 198 | 8.9 | 15q – losses | 161 | 7.2 |
| 17p + gains | 179 | 8.1 | 2q – losses | 153 | 6.8 |
| 16p + gains | 176 | 7.9 | Xp – losses | 152 | 6.8 |
| 8p + gains | 175 | 7.9 | Xq – losses | 126 | 5.7 |
| 15q + gains | 174 | 7.8 | 21q – losses | 122 | 5.5 |
| 5q + gains | 168 | 7.6 | Y – losses | 122 | 5.5 |
| 6q + gains | 164 | 7.4 | 18p – losses | 119 | 5.4 |
| 9q + gains | 156 | 7 | 19p – losses | 112 | 5 |
| 18p + gains | 153 | 6.9 | 17q – losses | 105 | 4.7 |
| 16q + gains | 140 | 6.3 | 3q – losses | 102 | 4.6 |
| 18q + gains | 136 | 6.1 | 12q – losses | 98 | 4.4 |
| 22q + gains | 133 | 6 | 19q – losses | 96 | 4.3 |
| 10p + gains | 131 | 5.9 | 1q – losses | 89 | 4 |
| Xq + gains | 129 | 5.8 | 6p – losses | 84 | 3.8 |
| 4q + gains | 118 | 5.3 | 16p – losses | 83 | 3.7 |
| 10q + gains | 117 | 5.3 | 5p – losses | 83 | 3.7 |
| 9p + gains | 116 | 5.2 | 2p – losses | 82 | 3.7 |
| Xp + gains | 115 | 4.7 | 8q – losses | 64 | 2.9 |
| 3p + gains | 104 | 4.7 | 7q – losses | 56 | 2.5 |
| 21q + gains | 101 | 4.5 | 20q – losses | 53 | 2.4 |
| 11p + gains | 97 | 4.4 | 20p – losses | 53 | 2.4 |
| 4p + gains | 95 | 4.3 | 12p – losses | 52 | 2.3 |
| Y + gains | 55 | 2.5 | 7p – losses | 50 | 2.3 |
| 14p + gains | 24 | 1.1 | 22p – losses | 36 | 1.6 |
| 21p + gains | 22 | 1 | 15p – losses | 21 | 0.9 |
| 13p + gains | 17 | 0.8 | 14p – losses | 10 | 0.5 |
| 15p + gains | 9 | 0.4 | 13p – losses | 9 | 0.4 |
| 22p + gains | 6 | 0.3 | 21p – losses | 3 | 0.1 |
| Total gains | 9320/2210 | 4.2 per tumour | Total losses | 6988/2210 | 3.1 per tumour |

tumours (mean 4.2 gain per tumour vs 3.1 loss per tumour). A variable pattern of chromosomal gain was observed, with the highest frequency of gain found in 8q (27.7%) and 1q (25.1%) (Table 1). This contrasts with chromosome 22p (0.3%) and 15p (0.4%) where gain of chromosomal material was rarely observed (Table 1). The most common regions of chromosomal loss were found on 13q (16.3% of all tumours), 9p (16.1%) and 8p (15.0%) (Table 1). Loss of chromosomal material was rarely seen on chromosome 21p (0.1%), 13p (0.4%) and 14p (0.5%). From Figure 2 it can be seen that levels of loss and gain are not uniform across all chromosomal regions. Certain chromosomal regions, such as 8q, are often gained (27.7%) but rarely lost (2.9%). Similarly, loss in chromosome 4q was more common (13.4%) than gain (5.3%). This pattern was not seen for all chromosomes, with loss of 13q (16.3%) only 1.8 times more common than gain (9.2%). Patterns of nearly equal frequency of loss and gain were also observed for chromosomes 14q (9% gain vs 8.2% loss) and 15q (7.8% gain vs 7.2% loss). However, this does not take into account the specific region of a chromosomal arm to which the genetic loss or gene amplification in solid tumours is mapped. It also does not account for tumour-specific patterns of chromosomal gain and loss (Table 2), where the same chromosomal arm is rarely lost and gained to an equal extent for a particular tumour type.

 © Cancer Research Campaign 1999

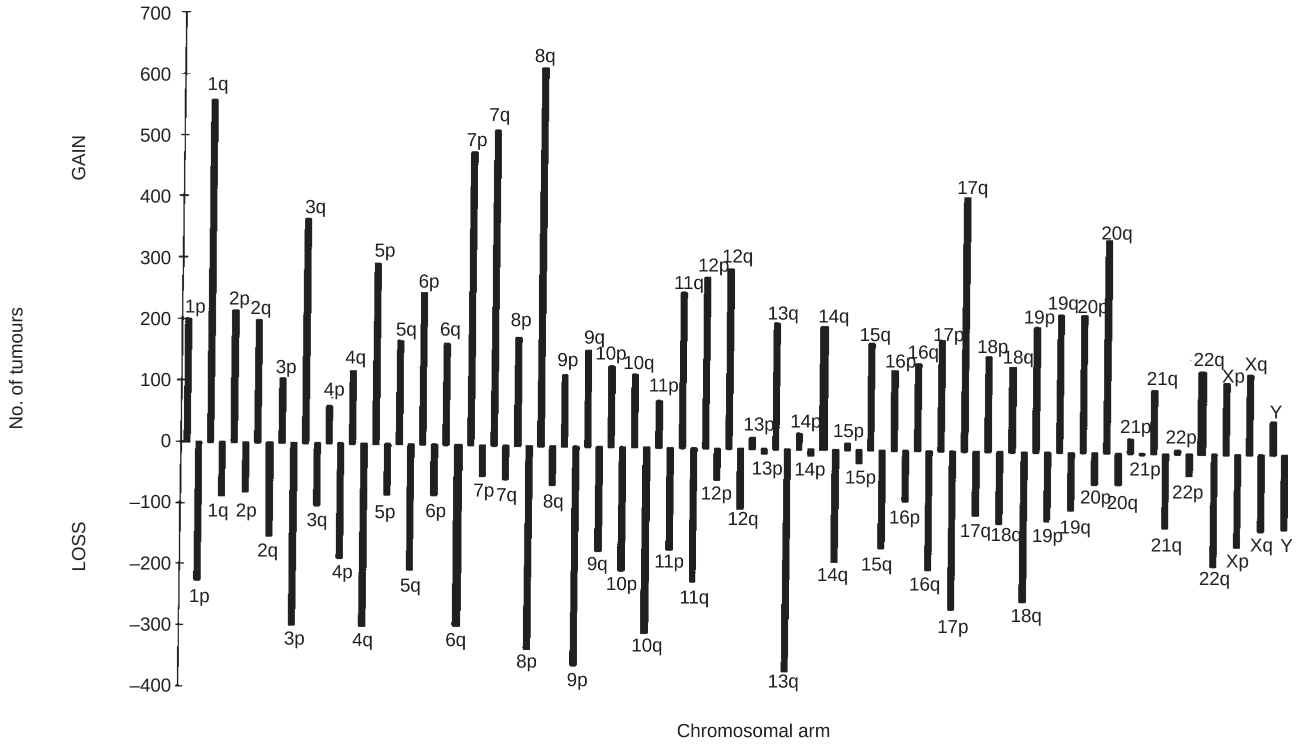

**Figure 2** The overall number of gains and losses detected in 2210 solid tumours from 27 different tumour types

### Specific tumour types

The frequency of chromosomal loss and gain varied between the individual tumour types, ranging from multiple regions per tumour (average gains: head and neck 12.2 per tumour, testicular 8.2 per tumour; loss: liver 7.5 per tumour, prostate 4.5 per tumour) to relatively rare events (average gains: neuroblastoma 0.5 per tumour, Wilms' 1.6 per tumour; loss: sarcoma 0.8 per tumour, Wilms' 1.3 per tumour (Table 3). The specific chromosomal regions of loss and gain differ substantially between specific tumour types. For example, gain in chromosome 12p occurred in 96.3% of testicular cancers and 0% of renal cancers (Table 2). New information on chromosomal loss or gain (Table 1) can be further specified amongst the various tumour types. For example, gain in chromosome 8q occurred in 27.7% of all tumours evaluated. However, on closer examination, frequency of 8q gain was high in tumours of the testis (40.7%), ovary (42.8%) and endometrium (45.5%), but was rarely found in renal tumours (1.3%) and neuroblastoma (3.0%). There is no chromosomal arm which demonstrated a consistent pattern of gain for all tumour types. Similar findings were demonstrated for chromosomal loss. For instance, 9p was lost in 16.1% of all tumours, but varied from a high frequency event (cutaneous melanoma 58.2%, pancreas 50.1%, brain 36.3%) to low (colon 7.5%, gastric cancer 7%) depending on the tumour type (Table 2).

### Specific tumour subtypes

For several tumours, CGH analysis was available for multiple histological subtypes (Table 4). This allowed assessment of both the frequency at which loss and gain occurred and the extent to which each specific chromosomal arm is involved for each subtype.

### *Colon*

Information on genomic alterations in colon cancer was available for low- and high-grade adenoma, primary carcinomas, liver metastases, and also carcinomas for which replication error repair status was known (Table 4). Ried et al (1995) found the frequency and degree of genetic aberrations increases with progression from low-grade adenoma through high-grade adenoma to carcinoma (Table 4). For example, gain in chromosome 7p was 7.1% in low-grade adenoma, 33.3% in high-grade adenoma and 50% in carcinoma. Similarly, gain in chromosome 20q was not detected in low-grade adenoma, but was at 33.3% and 75% in high-grade adenoma and carcinoma respectively. The frequency of alterations also increased with tumour progression: 3/47 chromosomal arms in low-grade adenoma, 21/47 high-grade adenoma, 32/47 carcinomas. A separate study by Paredes-Zaglul et al (1998) comparing primary carcinomas and liver metastases from patients with colorectal cancer found that the frequency of alteration remained constant at ~ 35/47 chromosomal arms between these two stages. However, a change was noted in the extent to which these arms were involved. The most obvious change being the increase in loss of genetic material between primary tumour and liver metastases. For example, loss at 8p was 30% in primary carcinomas compared with 80% in metastases. Similarly, loss of 18q was found in 50% of primary cases, but 90% of liver metastases. Changes in gain did not always follow the same pattern seen for loss. An increase in genetic instability was seen for some chromosomal regions in the transition from primary to metastases (e.g. 13q was gained in 30% of primary tumours compared with 50% in metastases). However, this was not the case for other regions, such as 12q, which was gained in 20% of primary carcinomas, but was normal in liver metastases. A difference in genetic instability was also seen between tumours with intact mismatch repair genes compared to

*© Cancer Research Campaign 1999*

**Table 2** The ten most frequently lost and gained chromosomal regions for each tumour type studied. Percentage of tumours with alterations of the specific chromosomal arm are shown. Fewer than ten altered arms were found in MCC.

| CR | GC | CR | H&N | CR | Pancreas | CR | Colon | CR | Prostate | CR | Testicular | CR | Breast | CR | Ovary | CR | Endo | CR | Cervical | CR | C mel | CR | MCC | CR | U mel | CR | Renal |
|---|---|---|---|---|---|---|---|---|---|---|---|---|---|---|---|---|---|---|---|---|---|---|---|---|---|---|---|
| **Top 10 losses** | | | | | | | | | | | | | | | | | | | | | | | | | | | |
| Y | 22.1 | 3p | 53.2 | 9p | 50.1 | 18q | 31.3 | 8p | 42 | 13q | 33.3 | 17p | 18.2 | 8p | 26.6 | 15q | 21.2 | 3p | 50 | 9p | 58.2 | 10q | 33.3 | 3p | 45.5 | 3p | 39.1 |
| 4q | 16.2 | 5q | 42.6 | 18q | 47.8 | 17p | 22.5 | 16q | 39 | 4q | 26 | 11q | 12.3 | 17p | 16.7 | 18q | 12.1 | 2q | 33.3 | 10q | 44.8 | 11q | 33.3 | 3q | 45.5 | 13q | 27.2 |
| 19p | 14.7 | 4p | 40.4 | 6q | 33.3 | 18p | 15 | 16q | 39 | 21q | 26 | 8p | 11.7 | 16q | 16.3 | 4q | 12.1 | 6q | 26.6 | 10p | 28.3 | | | 6q | 45.4 | 8p | 20.5 |
| 1p | 13.2 | 4q | 32 | 21q | 30.4 | 4p | 12.5 | 16q | 39 | 6q | 22.2 | 6q | 11.2 | 18q | 14.8 | 16q | 9.1 | 13q | 26.6 | 9q | 25.4 | | | 9p | 27.3 | 6q | 23.2 |
| 9p | 10.3 | 13q | 30 | 3p | 26.1 | 17q | 12.5 | 13q | 36 | 5q | 18.5 | 13q | 10.7 | 17q | 12.8 | 9p | 9.1 | 4p | 23.3 | 6q | 25.3 | | | 11p | 27.3 | 1q | 17.9 |
| 5q | 8.8 | 9p | 27.7 | 4q | 21.7 | 8p | 11.3 | 6q | 23 | Xq | 18.5 | 4p | 9.6 | 5q | 9.9 | 8p | 9.1 | 4q | 23.3 | 8p | 22.3 | | | 12p | 27.3 | 10q | 17.2 |
| 6p | 7.4 | 6q | 23.4 | 8p | 20.3 | 5q | 8.8 | 17p | 23 | 5q | 14.8 | 18q | 9.1 | Xp | 9.4 | Xp | 6.1 | 8p | 23.3 | 5q | 13.4 | | | 1p | 18.2 | 14q | 17.2 |
| 10q | 7.4 | 2q | 21.3 | 17p | 20.3 | 1p | 7.5 | 2q | 11 | 18p | 14.8 | 18p | 8 | Xq | 8.9 | 4p | 6.1 | 9p | 20 | 1p | 11.9 | | | 16q | 18.2 | 8p | 15.9 |
| 11q | 7.4 | 16q | 21.3 | 11q | 17.4 | 9p | 7.5 | 9q | 8 | 18q | 14.8 | 11p | 7.5 | 19p | 7.4 | 5q | 6.1 | 11q | 20 | 3q | 11.9 | | | Xp | 18.2 | 9p | 15.9 |
| 4p | 5.9 | 1p | 17 | 13q | 17.4 | 4q | 6.3 | 17q | 8 | 4p | 11.1 | 3p | 6.4 | 19q | 7.4 | 13q | 6.1 | 14q | 20 | 17p | 5.9 | | | Xq | 18.2 | 2p | 14.6 |
| **Top 10 gains** | | | | | | | | | | | | | | | | | | | | | | | | | | | |
| 17q | 42.6 | 3q | 61.7 | 20q | 47.8 | 20q | 33.8 | 8q | 43 | 12p | 96.3 | 1q | 52.9 | 8q | 42.8 | 1q | 54.5 | 3q | 76.6 | 8p | 41.8 | 1p | 66.6 | 8q | 63.6 | 17q | 23.2 |
| 8q | 30.9 | 5p | 59.6 | 8q | 34.8 | 7p | 23.8 | Xp | 26 | Xp | 60 | 8q | 38.5 | 20q | 38 | 8q | 45.5 | 1q | 46.6 | 7q | 37.3 | 1q | 66.6 | 6p | 54.5 | 7p | 18.5 |
| 20q | 30.9 | 2q | 51.1 | 11q | 29 | 13q | 27.5 | 7q | 23 | 8q | 40.7 | 17q | 19.8 | 3q | 33 | 10p | 21.2 | 5p | 30 | 7p | 35.8 | 6p | 66.6 | 8p | 18.2 | 5q | 17.2 |
| 17p | 27.9 | 11q | 44.7 | 7p | 27.5 | 7q | 22.5 | Xq | 20 | 7q | 30 | 11q | 19.8 | 1q | 23.6 | 10q | 15.2 | 6p | 26.6 | 8q | 30 | 6q | 66.6 | 17p | 18.2 | 1q | 16.6 |
| 7p | 26.5 | 17p | 44.7 | 7q | 27.5 | 8q | 23.8 | 7p | 18 | 8p | 30 | 16p | 13.9 | 1p | 20.2 | 13q | 21.2 | 20p | 23.3 | 6p | 30 | 18q | 66.6 | 17q | 18.2 | 7q | 15.9 |
| 20p | 26.5 | 17q | 44.7 | 20p | 27.5 | 20p | 17.5 | 8p | 14 | 7p | 25.9 | 3q | 10.2 | 7q | 19.7 | 5p | 27.3 | 8q | 20 | 1q | 25.4 | 20p | 66.6 | 7p | 9.1 | 17p | 15.2 |
| 1q | 23.5 | 15q | 42.6 | 14q | 26.1 | 9q | 13.8 | 3q | 12 | 20q | 22.2 | 22q | 10.2 | 2q | 18.7 | 6p | 33.3 | 9p | 20 | 17q | 15 | 20q | 66.6 | 9p | 9.1 | 19p | 12.6 |
| 9q | 19.1 | 20q | 42.6 | 18p | 26.1 | 9p | 7.5 | 18q | 12 | Xq | 22.2 | Xq | 9.6 | 11q | 18.2 | 11q | 24.2 | 15q | 20 | 20q | 15 | 8p | 33.3 | 9q | 9.1 | 16p | 11.3 |
| 13q | 19.1 | 7q | 40.4 | 3q | 20.3 | 1q | 6.3 | 2q | 10 | 2p | 18.5 | 8p | 5.3 | 6p | 16.7 | 7q | 21.2 | 19q | 20 | 2p | 13.4 | 8q | 33.3 | 11p | 9.1 | 22q | 8.6 |
| 3q | 17.6 | 12q | 40.4 | 12p | 17.4 | 13p | 6.3 | 16p | 9 | 2q | 18.5 | 3q | 5.3 | 13q | 11.3 | 6q | 9.1 | Xq | 20 | 5p | 11.9 | 9p | 33.3 | 11q | 9.1 | 20q | 7.9 |

| CR | Bladder | CR | Wilms' | CR | Sarcoma | CR | Rehab | CR | Lung | CR | Liver | CR | Neuro | CR | Brain | CR | GE* | CR | Para | CR | Pit | CR | N Endo | CR | GISTS |
|---|---|---|---|---|---|---|---|---|---|---|---|---|---|---|---|---|---|---|---|---|---|---|---|---|---|
| **Top 10 losses** | | | | | | | | | | | | | | | | | | | | | | | | | |
| 9q | 33.3 | 4q | 50 | 1q | 15.6 | 16q | 20.8 | 3p | 32.7 | 4q | 81.4 | 1p | 27.9 | 10q | 45.5 | 5q | 53.3 | 11q | 34 | 18p | 17.4 | 11q | 30 | 14q | 75 |
| 9p | 25 | 9p | 50 | 13q | 8.4 | 10p | 20.3 | 4q | 25.5 | 8p | 74.4 | 3p | 22.5 | 9p | 36.3 | 4p | 46.7 | 11p | 28.3 | 19p | 13 | 16p | 30 | 15q | 34 |
| 8p | 20.1 | 3q | 37.5 | 1p | 7.2 | 15q | 16.7 | 17p | 22.4 | 6q | 46.5 | 4p | 22.4 | 10p | 33.5 | 4q | 40 | 1p | 18.9 | 19q | 13 | 18p | 25 | 22q | 34 |
| 11q | 20 | 3p | 25 | 2q | 6.6 | 16p | 16.7 | 10q | 22.3 | 13q | 41.9 | 11q | 21.7 | 13q | 28 | 9p | 20 | 13q | 18.9 | 17p | 8.7 | 1p | 20 | 1p | 28.1 |
| 7q | 18.3 | 4p | 25 | 11q | 5.4 | 10q | 12.5 | 10p | 21.8 | Xq | 41.9 | 3q | 19.4 | 22q | 19.3 | 18q | 20 | 15q | 17 | 17q | 8.7 | 9p | 20 | 13q | 18.8 |
| 2q | 17.7 | 11p | 25 | 9p | 4.8 | 11q | 12.5 | 5q | 21.7 | 1p | 35 | 4q | 18.6 | 14q | 16.9 | 3p | 13.3 | 1q | 11.3 | 18q | 8.7 | 17p | 20 | 3p | 12.5 |
| Y | 16.6 | 11q | 25 | 4q | 4.2 | 14q | 12.5 | 13q | 21.4 | Xp | 34.9 | 10q | 14 | 6q | 16 | 5p | 13.3 | 6q | 11.3 | 1p | 4.3 | 19p | 15 | 8p | 6.3 |
| 11p | 14.6 | 17p | 25 | 3q | 3 | 1q | 8.3 | 9p | 21 | 17p | 30.2 | 21q | 21.7 | 18q | 12.1 | 6q | 13.3 | 9p | 11.3 | 2q | 4.3 | 19q | 15 | 1q | 3.1 |
| 10q | 12.5 | 19q | 25 | 8p | 3 | 2q | 8.3 | 20q | 13 | 16p | 25.6 | 8p | 11.6 | Xq | 10.2 | 1p | 6.7 | 9q | 9.4 | 8q | 4.3 | 4q | 10 | 3q | 3.1 |
| 16q | 11.5 | 20p | 25 | 10p | 3 | 4p | 8.3 | 8p | 12.3 | 14q | 20.9 | 11p | 11.6 | 19p | 7.5 | 2p | 6.7 | 2p | 5.7 | 13q | 4.3 | 6q | 10 | 5q | 3.1 |
| **Top 10 gains** | | | | | | | | | | | | | | | | | | | | | | | | | |
| 8q | 17.7 | 12p | 62.5 | 1q | 13.8 | 12q | 54.2 | 5p | 40.7 | 8q | 70 | 7p | 40.3 | 7q | 52 | 7q | 60 | 16p | 11.3 | 5q | 26.1 | 14q | 55 | 5q | 25 |
| 17q | 14.6 | 12q | 62.5 | 1p | 13.1 | 2p | 50 | 3q | 40.2 | 1q | 67.4 | 7q | 38 | 7p | 51 | 8q | 60 | 19p | 9.4 | 4q | 21.7 | 5p | 50 | 5p | 21.9 |
| 3q | 12.5 | 7p | 37.5 | 8q | 12.6 | 13q | 45.8 | 1q | 33.8 | 17q | 37.2 | 2p | 33.3 | 19p | 28 | 7p | 46.7 | 19q | 7.5 | 9p | 21.7 | 5q | 50 | 8q | 21.9 |
| 7p | 10.4 | 16q | 37.5 | 7q | 12 | 8q | 33.3 | 8q | 28.5 | 6p | 34.9 | 17p | 21 | 19q | 21.5 | 17q | 40 | 1p | 5.7 | 13q | 21.7 | 7p | 50 | 3q | 18.8 |
| 5p | 9.4 | 8p | 25 | 9p | 11.8 | 12p | 33.3 | 20p | 20.3 | 20q | 21 | 11q | 9.3 | 20q | 18.5 | 20q | 40 | 1q | 5.7 | Xq | 21.7 | 7q | 50 | 19p | 18.8 |
| 6p | 9.4 | 8q | 25 | 11q | 11.8 | 17q | 29.2 | 7p | 20 | 2q | 16.3 | 12q | 17.8 | 20p | 12.9 | 8p | 33.3 | 5q | 5.7 | 17q | 17.4 | 17q | 50 | 19q | 18.8 |
| 13q | 9.4 | 1q | 12.5 | 12q | 11.4 | 2q | 25 | 17q | 19.8 | 3q | 14 | 1q | 21.7 | 1p | 10.8 | 5q | 26.7 | 16q | 5.7 | Xp | 17.4 | 4p | 45 | 3p | 12.5 |
| 10p | 8.3 | 2p | 12.5 | 4p | 8.4 | 8p | 25 | 19q | 18.9 | 7p | 14 | 18q | 19.4 | 11q | 8.9 | 9q | 26.7 | 2p | 1.9 | 1q | 13 | 18q | 40 | 1q | 9.4 |
| 20q | 8.3 | 2q | 12.5 | 5p | 8.4 | 7q | 20.8 | 20q | 15.7 | 20p | 14 | 13q | 14 | 14q | 8 | 15q | 26.7 | 3q | 1.9 | 2p | 13 | 4q | 35 | 1p | 6.3 |
| 12q | 7.3 | 4p | 12.5 | 6q | 8.4 | 17p | 20.8 | 11q | 13.1 | 7q | 11.6 | 2q | 13.2 | Xq | 7.7 | 20p | 26.7 | 5p | 1.9 | 2q | 13 | 12q | 35 | 8p | 6.3 |

CR = Chromosomal region; C mel = Cutaneous melanoma; Endo = Endometrial; H&N = Head and neck; MCC = Merkel cell carcinoma; Neuro = Neuroblastoma; Rhab = Rhabdomyosarcoma; GC = Gastric carcinoma; U mel = Uveal melanoma; OSCC = Oral squamous cell carcinoma; GISTS = Gastrointestinal stromal tumours; GE = Gastro-oesophageal adenocarcinoma; Para = Parathyroid adenomas; Pit = Non-functioning pituitary; N endo = Sporadic neuroendocrine tumours of the digestive system. *No distinction was made between oesophageal adenocarcinoma from Barrett's mucosa and gastric adenoacarcinoma arising at the fundus of the stomach. As a result, CGH data for GC and GE could not be merged.

© Cancer Research Campaign 1999

**Table 3** The number of altered chromosomal arms observed among the different tumour types

| Cancer type | Gains/tumour | | Losses/tumour | | Total instability (loss + gain) per tumour |
|---|---|---|---|---|---|
| Gastric carcinoma | 365\68 | 5.4 | 128\68 | 1.9 | 7.3 |
| Gastrointestinal stromal | 52\32 | 1.6 | 71\32 | 2.2 | 3.8 |
| Head and neck | 588\47 | 12.5 | 245\47 | 5.2 | 17.7 |
| Pancreatic | 231\51 | 4.5 | 188\51 | 3.7 | 8.2 |
| Colorectal | 204\80 | 2.6 | 190\80 | 2.4 | 5 |
| Prostate | 312\100 | 3.1 | 447\100 | 4.5 | 7.6 |
| Testicular | 337\41 | 8.2 | 171\41 | 4.2 | 12.4 |
| Breast | 752\187 | 4 | 549\187 | 2.9 | 6.9 |
| Ovarian | 1136\203 | 5.6 | 499\203 | 2.5 | 8.1 |
| Endometrial | 186\33 | 5.6 | 50\33 | 1.5 | 7.1 |
| Cervical | 163\30 | 5.4 | 124\30 | 4.1 | 9.5 |
| Cutaneous melanoma | 203\67 | 3 | 227\67 | 3.4 | 6.4 |
| Merkel cell carcinoma | 23\3 | 8 | 13\3 | 4.3 | 12.3 |
| Uveal melanoma | 23\11 | 2.1 | 27\11 | 2.5 | 4.6 |
| Renal | 346\151 | 2.3 | 530\151 | 3.5 | 5.8 |
| Bladder | 222\96 | 2.3 | 278\96 | 2.9 | 5.2 |
| Wilms' | 89\54 | 1.6 | 71\54 | 1.3 | 2.9 |
| Connective tissue sarcoma | 530\193 | 2.7 | 154\193 | 0.8 | 3.5 |
| Rhabdomyosarcoma | 158\24 | 6.6 | 61\24 | 2.5 | 9.1 |
| Lung | 845\142 | 6 | 599\142 | 4.2 | 10.2 |
| Liver | 201\43 | 4.7 | 322\43 | 7.5 | 12.2 |
| Neuroblastoma | 56\118 | 0.5 | 439\118 | 3.7 | 4.2 |
| Brain | 1152\325 | 3.5 | 1076\325 | 3.3 | 6.8 |
| Gastro-oesophageal | 100\15 | 6.7 | 50\15 | 3.3 | 10 |
| Parathyroid | 38\53 | 7.2 | 121\53 | 2.3 | 9.5 |
| Pituitary | 92\23 | 4 | 22\53 | 4.2 | 8.2 |
| Neuroendocrine* | 162\20 | 8.1 | 57\20 | 2.9 | 11 |

*Sporadic neuroendocrine tumours of the digestive system.

those with deficient repair ability (Table 4). As expected, the tumours lacking repair function had a higher frequency of instability. For example, gain of 7p and 7q was seen in 33% of tumours with non-functioning repair genes, while these aberrations were absent in tumours with intact DNA repair phenotype. Although a relationship between genomic instability and both tumour progression and repair deficiency had been previously suggested, CGH has provided strong data to support this hypothesis in tumour specimens.

### *Ovary*

Several studies have been published assessing the genomes of ovarian cancer cases. The available data were split into ovarian cancers derived from the epithelia and those derived from germ cells. Cancers of the epithelia were then further subdivided into sporadic and hereditary cases. The hereditary cases were defined as such based on BRCA1 and BRCA2 status. It is appreciated that some papers did not assess their cases for BRCA1 and BRCA2 and that a small percentage of the sporadic cases may have altered BRCA genes. Overall, however, this division of ovarian tumours has yielded some useful observations. Firstly, it was found that the frequency of genetic aberrations was greatest in the sporadic cases at 41/47 chromosomal arms, compared with 33/47 in hereditary cases and 30/47 in the germ cell tumours. The greatest level of concordance was at 1q and 8q where gains occurred at approximately 30% and 50%, respectively, in all three tumour types. Both hereditary and sporadic cases had a high degree of gain at 3q (40.6% in sporadic and 50% in inherited cases). This is in contrast to the same region being gained in only 5.3% of germ cell tumours. However, all three tumour subtypes are likely to have some common genetic origin based on the observation that regions such as 1q and 8q are gained to an equal extent in all ovarian cancer types so far studied by CGH.

### *Prostate*

The data on prostate cancer allowed comparison of CGH results in patient cohorts with primary resected carcinomas or tumours that recurred after hormone therapy. It has been speculated that further genetic damage allows a subclone of tumour cells to acquire resistance to chemotherapy and such studies can test this hypothesis. Very little change in the frequency of genetic aberration between primary carcinoma and recurrent carcinoma was seen (39/47 in primary vs 42/47 in recurrent). However, differences were seen in the degree of genetic aberration when specific chromosomal regions were considered. For example, gain in chromosome 8q was seen in 25.9% primary carcinomas compared with 73.9% in recurrent cases. Similarly, 19p was lost in 3.7% of primary tumours and 34.8% in recurrent cases. Gain in the region containing the androgen receptor gene, Xp, increased from 7.4% in primary tumour to 28.3% in patients with recurrent disease. This is consistent with androgen receptor gene amplification as a mechanism of resistance to hormone therapy. However, this was not always the case with some regions of the genome only slightly changed in the degree of the aberration between primary and recurrent. For example, 3p was lost in 1.9% of primary tumours and 4.3% in recurrent cases. Generally, the data support the hypothesis that increased tumour aggression is the phenotype of a more unstable genome.

*© Cancer Research Campaign 1999*

**Table 4** Patterns of loss(−) and gain in specific tumour subtypes shown as the percentage of tumours with involvement for selected chromosomes

| **Tumour type** | **Colon†** | | | | | | |
|---|---|---|---|---|---|---|---|
| **CR** | **Iga *n*=14** | **Hga *n*=12** | **Carcinoma *n*=16** | **Min– *n*=6** | **Min+ *n*=12** | **Primary *n*=10** | **Metastases *n*=10** |
| 7p | 7.1 | 33.3 | 50 | 0 | 33.3 | 10 | 10 |
| 7q | 0 | 25 | 31.3 | 0 | 33.3 | 30 | 30 |
| 8p | 0 | 0 | 0 | 0 | 0 | 10&–30 | 10&–80 |
| 12q | 0 | 8.3 | 6.3 | 0 | 0 | 20 | 0 |
| 13q | 0 | 8.3 | 50 | –16.7 | 41.7 | 30&–10 | 50 |
| 18q | 0 | –16.7 | –37.5 | 0 | –25 | –50 | –90 |
| 20q | 0 | 33.3 | 75 | 0 | 25 | 50 | 40 |
| Involved arms | 3\47 | 21\47 | 32\47 | 3\47 | 22\47 | 34\47 | 35\47 |

| **Tumour type** | **Ovary** | | |
|---|---|---|---|
| **CR** | **Sporadic* *n*=148** | **Inherited *n*=20** | **OGCT *n*=19** |
| 1q | 34.8&–0.7 | 30 | 31.6 |
| 2q | 18.1&–1.4 | 50&–5 | 0 |
| 3q | 40.6 | 50 | 5.3&–5.3 |
| 8q | 52.9&–0.7 | 55 | 42.1 |
| 21q | 5.8&–7.2 | 0 | 47.4 |
| Involved arms | 41\47 | 33\47 | 30\47 |

| **Tumour type** | **Prostate** | |
|---|---|---|
| **CR** | **Primary *n*=54** | **Recurrent *n*=46** |
| 3p | –1.9 | 4.3&–4.3 |
| 7p | 3.7 | 34.8&–2.2 |
| 7q | 13&–1.9 | 34.8&–2.2 |
| 8p | –46.5 | 8.7&–60.9 |
| 8q | 25.9 | 73.9 |
| 19p | 7.4&–3.7 | –34.8 |
| Xp | 7.4&–1.9 | 28.3&–8.7 |
| Xq | 14.8 | 15.2&–6.5 |
| Involved arms | 39\47 | 42\47 |

| **Tumour type** | **Sarcoma** | | | | | |
|---|---|---|---|---|---|---|
| **CR** | **Osteosarcoma *n*=14** | **RMS-E *n*=10** | **RMS-A *n*=14** | **Liposarcoma *n*=14** | **ASPS *n*=13** | **Ewing's *n*=20** |
| 2p | 0 | 50 | 50 | 0 | 0 | 5 |
| 6p | 28.6 | 0 | 7.1 | 0 | 0 | 10 |
| 2q | 7.1 | 60 | –14.3 | 14.3 | 0 | 5 |
| 13q | 14.3 | 60&–10 | 35.7&–7.1 | 7.1&–21.4 | 0 | 5 |
| 16q | 0 | 2&–30 | 7.1&–7.1 | 7.1 | –7.7 | 5&–5 |
| Involved arms | 19\47 | 38\47 | 35\47 | 38\47 | 14\47 | 28\47 |

In several tumour subtypes both loss and gain were observed on the same chromosomal arm. Variation in the number of chromosomal arms involved in genetic instability was also observed between subtypes. *Contains tumours which were not evaluated for BRCA1 and BRCA2 status. † represents data from three separate studies evaluating tumour progression, microsatellite instability and metastasis respectively. CR = chromosomal region; Iga = low-grade adenoma; Hga = high-grade adenoma; OGCT = ovarian germ cell tumours; MIN+ = without microsatellite instability; MIN– = with microsatellite instability; RMS-E = rhabdomyosarcoma embryonal; RMS-A = rhabdomyosarcoma alveolar; ASPS = alveolar soft part sarcoma

### *Connective tissue tumours*

CGH data were available for several tumour types (liposarcoma, alveolar soft part sarcoma, osteosarcoma, Ewing's, rhabdomyosarcoma and osteochondroma). Unlike the other subtypes discussed (colon, ovary and prostate), tumours of the connective tissue are found in many different sites throughout the body. Considering the frequency of genetic aberration, the widest range of variation between subtypes among any tumour type in the literature is observed in the sarcomas. At one end of the spectrum a study on osteochondromas reports no genetic aberrations in 15 cases of this benign tumour type (Larramendy et al, 1997). Such a paper is unique in the CGH literature as all other investigations report some genomic change detectable by CGH. The alveolar soft part- and osteosarcomas show low to moderate frequency of genetic aberration at 14 and 19 out of 47 chromosomal arms respectively. While the other subtypes showed moderate to high numbers of arms involved (range 28–38 of 47). Another unique observation in the CGH literature was seen in an osteosarcoma study where only gain of genetic material was detected (Forus et al, 1995). Caution must be exercised when interpreting such results as it is unlikely that this cancer is the exception where no loss of genetic material is required for its development. More likely any loss, such as that of a tumour suppressor gene, is below detection by CGH. Rhabdomyosarcomas are further subdivided histologically into alveolar and embryonal types. Generally, a higher degree of gain and loss is seen in the embryonal rhabdomyosarcoma compared

*© Cancer Research Campaign 1999*

with alveolar rhabdomyosarcoma (Weber-Hall et al, 1996). For example, a sub-chromosomal region of 13q is gained in 60% and lost in 10% of embryoneal, while the same region is gained in 35.7% and lost in 7.1% of alveolar, rhabdomyosarcomas. The exception is 2p, which is lost in 50% of cases in both subtypes. Comparing both subtypes of rhabdomyosarcoma with other sarcomas it is observed that a gain of 2q is not present in a high proportion in all sarcomas. In fact no change in 2q is detected in liposarcoma or alveolar soft part sarcoma and gain in Ewing's sarcoma is detected in less than 10% of all cases. This pattern of a certain chromosomal region commonly occurring in a specific subtype, but not in any other, continues for many chromosomal regions, suggesting that sarcomas are very distinct in terms of their genetic origin, with each subtype having its own marker chromosomal aberrations. This may be due to the variation in tissue type in which these tumours arise. No single chromosomal aberration was found to be present in a high proportion of all sarcomas.

### PATTERNS OF GENOMIC IMBALANCE OR INSTABILITY IN SOLID TUMOURS

The degree of genomic imbalance detectable by CGH differs significantly between the various solid tumours (Table 3). Chromosomal gain varied from 0.5 to 12.5 chromosome arms per tumour with a median of 4.5, while loss varied from 0.8 to 7.5 chromosomal arms per tumour with a median of 3.3. Total instability (chromosomal loss + chromosomal gain per number of tumours) was highest in head and neck tumours (17.7 lesions per tumour) and testicular (12.4 lesions per tumour) and lowest in Wilms' (2.9 lesions per tumour) and sarcoma (3.5 lesions per tumour) tumours. These frequencies represent an overall value for each specific tumour type, as information on the chromosomal alterations found within an individual tumour was not available in most literature reports of CGH in human solid tumours. Difference in the degree of loss or gain was also observed between the various solid tumours (Table 3). For example, chromosomal gain was observed more frequently than loss in the sarcomas and endometrial tumours, while loss was more frequently observed for renal and liver tumours. It is unknown whether these patterns represent coincidental changes from generalized genomic instability or suggest that some cancers are more likely to be influenced by the loss of tumour suppressor genes (genomic loss), while others are more frequently influenced by oncogene over expression (genomic gain). In addition, several studies have identified an association between the acquisition of genetic aberrations and patient survival (Iwabuchi et al, 1995; Tanner et al, 1995). However, there are discrepancies in this association found in Table 3, and any correlations between biological markers and patient survival need to be interpreted cautiously in the context of modern therapy.

### COMPARISON WITH SOLID TUMOUR KARYOTYPE ANALYSIS

Classical karyotyping of metaphase chromosomes has been successfully performed for some solid tumours. A recent review reported the frequencies and distribution of chromosomal imbalances detected in 3185 solid tumours from 11 tumour types using chromosomal banding (Merkel et al, 1997). Overall, deletions were more common than gains in this analysis. Our review has found the opposite, with gains more commonly detected by CGH than losses. This difference may reflect the difficulties with using tumour karyotyping to identify the chromosomal changes that have occurred in tumours with highly complex rearrangements and will be influenced to some extent by amplified segments being hidden among unidentified marker chromosomes. CGH should be more sensitive for the detection of the presence of gains than losses and therefore the discrepancies with the above study are likely to reflect technical limitations of the two methods. By restricting analysis to common alterations (i.e. the gain or loss was detected in at least 15% of the tumours studied for that particular tumour type), the classical karyotyping studies described fewer regions of gain and loss than CGH for every tumour type evaluated. CGH appeared to identify the same alterations described using the karyotyping approach (with the exception of balanced translocations which are not detectable by CGH), but also observed additional regions of loss or gain. For example, only two regions of gain were detected in ovarian carcinoma by traditional cytogenetic analysis compared with 26 regions of gain seen by CGH. However, there have been too few studies of solid tumour cytogenetics using both CGH and chromosome banding for any firm conclusions regarding concordance between the two techniques. Nevertheless, the accumulating body of evidence in the literature suggests that CGH is more sensitive than other current technologies available for global assessment of loss and/or gain in solid tumour genomes.

### CONCLUSION

From this review, it is apparent that no specific chromosomal imbalances are found in all cancers, with the most frequently identified regions of gain or loss occurring in 27.7% and 16.3% of tumours respectively. This reflects the heterogeneity in genomic alterations identified in different tumour types. In addition, much variation within tumour subtypes was observed.

The development of CGH has provided the technology to identify many new areas of genomic alteration which were not previously recognized to be altered in tumorigenesis. This has now expanded the number of areas of the genome for which more detailed molecular study is required to give a clearer more complete understanding of cancer biology.

Other areas where CGH could potentially make a significant contribution include its application in tumour diagnosis, as a prognostic tool, or for investigations into chemoresistance. The ability to assess the entire genome in a single experiment makes this technique potentially useful as an adjunct to routine histopathology. Several studies have established the feasibility of using CGH to detect genomic regions involved in the acquisition of resistance in human cancer cell lines and have detected novel regions of the genome not previously recognized to be involved in drug resistance (du Manoir et al, 1997; Wasenius et al, 1997; Leyland-Jones et al, 1998; Rooney et al, 1998). This provides the impetus to apply CGH to human tumour specimens in the context of modern drug therapy to assess its role in optimizing patient treatment.

### ACKNOWLEDGEMENTS

Many thanks to Dr Lars-Peter Erwig for translation of a German paper. This work was supported in part by a University of Aberdeen Research Consortium studentship, a University of Aberdeen Equipment Award and an Aberdeen Royal Infirmary endowment grant.

© Cancer Research Campaign 1999 

### REFERENCES

Dowsett M, Daffada A, Chan CMW and Johnston SRD (1997) Oestrogen receptor mutants and variants in breast cancer. *Eur J Cancer* **33**: 1177–1183
du Manoir S, Speicher MR, Joos S, Schrock E, Popp S, Döhner H, Kovacs G, Robert-Nicoud M, Lichter P and Cremer T (1993) Detection of complete and partial chromosome gains and losses by comparative genomic in situ hybridization. *Hum Genet* **90**: 590–610
du Manoir S, Myers TG, Paull KD, Bell DW, Liu ZM, Feder MM, Weinstein JN, Sonoda G and Testa JR (1997) Analysis by CGH of the tumor cell lines of NCI anticancer drug discovery screen. *Proc Am Assoc Cancer Res* **38**: 606
Forozan F, Karhu R, Kononen J, Kallioniemi A and Kallioniemi O-P (1997) Genome screening by comparative genomic hybridisation. *TIGS* **13**: 405–409
Forus A, Olde Weghuis D, Smeets D, Fodstad Ø, Myklebost O and van Kessel AG (1995) Comparative genomic hybridization analysis of human sarcomas: II. Identification of novel amplicons at 6p and 17p in osteosarcomas. *Genes Chromosomes Cancer* **14**: 15–21
Isola J, DeVries S, Chu L, Ghazvini S and Waldman F (1994) Analysis of changes in DNA sequence copy number by comparative genomic hybridization in archival paraffin-embedded tumor samples. *Am J Pathol* **145**: 1301–1308
Iwabuchi H, Sakamoto M, Sakunaga H, Ma Y-Y, Carcangiu ML, Pinkel D, Yang-Feng TL and Gray JW (1995) Genetic analysis of benign, low-grade, and high-grade ovarian tumors. *Cancer Res* **55**: 6172–6180
Kallioniemi A, Kallioniemi O-P, Sudar D, Rutovitz D, Gray JW, Waldman F and Pinkel D (1992) Comparative genomic hybridisation for the genetic analysis of solid tumours. *Science* **258**: 818–821
Kallioniemi O-P, Kallioniemi A, Sudar D, Rutovitz D, Gray JW, Waldman F and Pinkel D (1993) Comparative genomic hybridization: a rapid new method for detecting and mapping DNA amplification in tumors. *Semin Cancer Biol* **4**: 41–46
Kuukasjarvi T, Tanner M, Pennanen S, Karhu R, Visakorpi T and Isola J (1997) Optimizing DOP-PCR for universal amplification of small DNA samples in comparative genomic hybridization. *Genes Chromosomes Cancer* **18**: 94–101
Larramendy ML, Tarkkanen M, Blomqvist C, Virolainen M, Wiklund T, AskoSeljavaara S, Elomaa I and Knuutila S (1997) Comparative genomic hybridization of malignant fibrous histiocytoma reveals a novel prognostic marker. *Am J Pathol* **151**: 1153–1161
Levitzki A and Gazit A (1995) Tyrosine kinase inhibition: an approach to drug development. *Science* **267**: 1782
Leyland-Jones B, Bradshaw TD, Skelton L, Kelland LR, Fisher LM and Hiorns LR (1998) Genomic alterations associated with acquired resistance to novel antitumor agents. *Proc Am Assoc Cancer Res* **39**: 658
Mertens F, Johansson B, Höglund M and Mitelman F (1997) Chromosomal imbalance maps of malignant solid tumors: a cytogenetic survey of 3185 neoplasms. *Cancer Res* **57**: 2765–2780
Paredes-Zaglul A, Kang JJ, Essig YP, Mao WG, Irby R, Wloch M and Yeatman TJ (1998) Analysis of colorectal cancer by comparative genomic hybridization: evidence for induction of the metastatic phenotype by loss of tumor suppressor genes. *Clin Cancer Res* **4**: 879–886
Ried T, Knutzen R, Steinbeck R, Blegen H, Schrock E, Heselmeyer K, duManoir S and Auer G (1996) Comparative genomic hybridization reveals a specific pattern of chromosomal gains and losses during the genesis of colorectal tumors. *Genes Chromosomes Cancer* **15**: 234–245
Ried T, Liyanage M, du Manoir S, Heselmeyer K, Auer G, Macville M and Schröck E (1997) Tumor cytogenetics revisited: comparative genomic hybridisation and spectral karyotyping. *J Mol Med* **75**: 801–814
Rooney PH, Marsh S, Stevenson DAJ, Johnston PG, Haites NE, Cassidy J and McLeod HL (1998) Genome wide assessment in cell lines resistant to thymidylate synthase inhibitors. *Cancer Res* **58**: 5042–5045
Tanner MM, Tirkkonen M, Kallioniemi A, Holli K, Collins C, Kowbel D, Gray JW, Kallioniemi O-P and Isola J (1995) Amplification of chromosomal region 20q13 in invasive breast cancer: prognostic implications. *Clin Cancer Res* **1**: 1455–1461
Telenius H, Pelmear AH, Tunnacliffe A, Carter NP, Behmel A, Ferguson-Smith MA, Nordenskjold M, Pfragner R and Ponder BAJ (1992) Cytogenetic analysis by chromosome painting using DOP-PCR amplified flow-sorted chromosomes. *Genes Chromosomes Cancer* **4**: 257–263
Vogel F (1979) Genetics of retinoblastoma. *Hum Genet* **52**: 1–54
Waldman FM, Sauter G, Sudar D and Thompson CT (1996) Molecular cytometry of cancer. *Hum Pathol* **27**: 441–449
Wasenius VM, Jekunen A, Monni O, Joensuu H, Aebi S, Howell SB and Knuutila S (1997) Comparative genomic hybridization analysis of chromosomal changes occurring during development of acquired resistance to cisplatin in human ovarian carcinoma cells. *Genes Chromosomes Cancer* **18**: 286–291
Weber-Hall S, Anderson J, McManus A, Abe S, Nojima T, Pinkerton R, Pritchard-Jones K and Shipley J (1996) Gains, losses, and amplification of genomic material in rhabdomyosarcoma analyzed by comparative genomic hybridization. *Cancer Res* **56**: 3320–3224

### APPENDIX

### Bladder

Kallioniemi A, Kallioniemi O-P, Citro G, Sauter G, DeVries S, Kerschmann R, Caroll P and Waldman F (1995) Identification of gains and losses of DNA sequences in primary bladder cancer by comparative genomic hybridization. *Genes Chromosomes Cancer* **12**: 213–219
Richter J, Jiang F, Görög J-P, Sartorius G, Egenter C, Gasser TC, Moch H, Mihatsch MJ and Sauter G (1997) Marked genetic differences between stage pTa and stage pT1 papillary bladder cancer detected by comparative genomic hybridization. *Cancer Res* **57**: 2860–2864
Voorter C, Joos S, Bringuier P-P, Vallinga M, Poddighe P, Schalken J, du Manoir S, Ramaekers F, Lichter P and Hopman A (1995) Detection of chromosomal imbalances in transitional cell carcinoma of the bladder by comparative genomic hybridization. *Am J Pathol* **146**: 1341–1354

### Brain

Carlson KM, Bruder C, Nörderskjord M and Dumanski JP (1997) 1p and 3p deletions in meningiomas without detectable aberrations of chromosome 22 identified by comparative genomic hybridisation. *Genes Chromosomes Cancer* **20**: 419–424
Khan J, Parsa NZ, Harada T, Meltzer PS and Carter NP (1998) Detection of gains and losses in 18 meningiomas by comparative genomic hybridization. *Cancer Genet Cytogenet* **103**: 95–100
Kim DH, Mohapatra G, Bollen A, Waldman FM and Feuerstein BG (1995) Chromosomal abnormalities in glioblastoma-multiforme tumors and glioma cell-lines detected by comparative genomic hybridization. *Int J Cancer* **60**: 812–819
Mohapatra G, Bollen AW, Kim DH, Lamborn K, Moore DH, Prados MD and Feuerstein BG (1998) Genetic analysis of glioblastoma multiforme provides evidence for subgroups within the grade. *Genes Chromosomes Cancer* **21**: 195–206
Nishizaki T, Ozaki S, Harada K, Ito H, Arai H, Beppu T and Sasaki K (1998) Investigation of genetic alterations associated with the grade of astrocytic tumor by comparative genomic hybridization. *Genes Chromosomes Cancer* **21**: 340–346
Reardon DA, Michalkiewicz E, Boyett JM, Sublett JE, Entrekin RE, Ragsdale ST, Valentine MB, Behm FG, Li H, Heideman RL, Kun LE and Shapiro DN (1997) Extensive genomic abnormalities in childhood medulloblastoma by comparative genomic hybridization. *Cancer Res* **57**: 4042–4047
Sallinen SL, Sallinen P, Haapasalo H, Kononen J, Karhu R, Helen P and Isola J (1997) Accumulation of genetic changes is associated with poor prognosis in grade II astrocytomas. *Am J Pathol* **151**: 1799–1807
Schlegel J, Scherthan H, Arens N, Stumm G and Kiessling M (1996) Detection of complex genetic alterations in human glioblastoma multiforme using comparative genomic hybridization. *J Neuropath Exp Neuro* **55**: 81–87
Schröck E, Thiel G, Lozanova T, du Manoir S, Meffert M-C, Jauch A, Speicher MR, Nürnberg P, Vogel S, Jänisch W, Doris-Keller H, Ried T, Witkowski R and Cremer T (1994) Comparative genomic hybridization of human malignant gliomas reveals multiple amplification sites and nonrandom chromosomal gains and losses. *Am J Pathol* **144**: 1203–1218
Schröck E, Blume C, Meffert M-C, du Manior S, Bersch W, Kiessling M, Lozanowa T, Thiel G, Witkowski R, Ried T and Cremer T (1996) Recurrent gain of chromosome arm 7q in low-grade astrocytic tumors studied by comparative genomic hybridization. *Genes Chromosomes Cancer* **15**: 199–205
Weber RG, Sommer C, Albert FK, Kiessling M and Cremer T (1996) Clinically distinct subgroups of glioblastoma multiforme studied by comparative genomic hybridisation. *Lab Invest* **74**: 108–119
Weber RG, Sabel M, Reifenberger J, Sommer C, Oberstraβ J, Reifenberger G, Kiessling M and Cremer T (1996) Characterization of genomic alterations associated with glioma progression by comparative genomic hybridization. *Oncogene* **13**: 983–994

© Cancer Research Campaign 1999

### Breast

Courjal F and Theillet C (1997) comparative genomic hybridization analysis of breast tumors with predetermined profiles of DNA amplification. *Cancer Res* **57**: 4368–4377

Guan X-Y, Xu J, Anzick SL, Shang H, Trent JM and Meltzer PS (1996) Hybrid selection of transcribed sequences from microdissected DNA: isolation of genes within an amplified region at 20q11-q13.2 in breast cancer. *Cancer Res* **56**: 3446–3450

Isola J, Kallioniemi O-P, Chu LW, Fuqua SAW, Hilsenbeck SG, Osborne K and Waldman FM (1995) Genetic aberrations detected by comparative genomic hybridization predict outcome in node-negative breast cancer. *Am J Pathol* **147**: 905–911

Kuukasjärvi T, Tanner M, Pennanen S, Karhu R, Kallioniemi OP and Isola J (1997) Genetic changes in intraductal breast cancer detected by comparative genomic hybridization. *Am J Pathol* **150**: 1465–1471

Lu Y-J, Birdsall S, Osin P, Gusterson B and Shipley J (1997) Phyllodes tumors of the breast analyzed by comparative genomic hybridization and association of increased 1q copy number with stromal overgrowth and recurrence. *Genes Chromosomes Cancer* **20**: 275–281

Nishizaki T, DeVries S, Chew K, Goodson WH III, Ljung B-M, Thor A and Waldman FM (1997) Genetic alterations in primary breast cancers and their metastases: direct comparison using modified comparative genomic hybridization. *Genes Chromosomes Cancer* **19**: 267–272

Ried T, Just KE, Holtgreve-Grez H, du Manoir S, Speicher MR, Schröck E, Latham C, Blegen H, Zetterberg A, Cremer T and Auer G (1995) Comparative genomic hybridization of formalin-fixed, paraffin-embedded breast tumors reveals different patterns of chromosomal gains and losses in fibroadenomas and diploid and aneuploid carcinomas. *Cancer Res* **55**: 5414–5423

Tanner MM, Tirkkonen M, Kallioniemi A, Collins C, Stokke T, Karhu R, Kowbel D, Shadravan F, Hintz M, Kuo W-L, Waldman FM, Isola J, Gray JW and Kallioniemi O-P (1994) Increased copy number at 20q13 in breast cancer: defining the critical region and exclusion of candidate genes. *Cancer Res* **54**: 4257–4260

Tanner MM, Tirkkonen M, Kallioniemi A, Holli K, Collins C, Kowbel D, Gray JW, Kallioniemi O-P and Isola J (1995) Amplification of chromosomal region 20q13 in invasive breast cancer: prognostic implications. *Clin Cancer Res* **1**: 1455–1461

Tanner MM, Karhu RA, Nupponen NN, Borg A, Baldetorp B, Pejovic T, Ferno M, Killander D and Isola J (1998) Genetic aberrations in hypodiploid breast cancer: frequent loss of chromosome 4 and amplification of Cyclin D1 oncogene. *Am J Pathol* **153**: 191–199

Tirkkonen M, Tanner M, Karhu R, Kallioniemi A, Isola J and Kallioniemi OP (1998) Molecular cytogenetics of primary breast cancer by CGH. *Genes Chromosomes Cancer* **21**: 177–184

### Cervix

Heselmeyer K, Macville M, Schröck Blegen H, Hellström A-C, Shah K, Auer G and Ried T (1997) Advanced-stage cervical carcinomas are defined by a recurrent pattern of chromosomal aberrations revealing high genetic instability and a consistent gain of chromosome arm 3q. *Genes Chromosomes Cancer* **19**: 233–240

### Colon

Nakao K, Shibusawa M, Tsunoda A, Yoshizawa H, Murakami M, Kusano M, Uesugi N and Sasaki K (1998) Genetic changes in primary colorectal cancer by comparative genomic hybridization. *Surg Today-Jn J Surg* **28**: 567–569

Paredes-Zaglul A, Kang JJ, Essig YP, Mao WG, Irby R, Wloch M and Yeatman TJ (1998) Analysis of colorectal cancer by comparative genomic hybridization: evidence for induction of the metastatic phenotype by loss of tumor suppressor genes. *Clin Cancer Res* **4**: 879–886

Ried T, Knutzen R, Steinbeck R, Blegen H, Schrock E, Heselmeyer K, duManoir S and Auer G (1996) Comparative genomic hybridization reveals a specific pattern of chromosomal gains and losses during the genesis of colorectal tumors. *Genes Chromosomes Cancer* **15**: 234–245

Schlegel J, Stumm G, Scherthan H, Bocker T, Zirngibl H, Rüschoff J and Hofstädter F (1995) Comparative genomic in situ hybridization of colon carcinomas with replication error. *Cancer Res* **55**: 6002–6005

### Cutaneous melanoma

Bastian BC, Leboit PE, Hamm H, Brocker EB and Pinkel D (1998) Chromosomal gains and losses in primary cutaneous melanomas detected by comparative genomic hybridization. *Cancer Res* **58**: 2170–2175

Wiltshire RN, Duray P, Bittner ML, Visakorpi T, Meltzer PS, Tuthill RJ, Liotta LA and Trent JM (1995) Direct visualization of the clonal progression of primary cutaneous melanoma: application of tissue microdissection and comparative genomic hybridization. *Cancer Res* **55**: 3954–3957

### Endometrium

Pere H, Tapper J, Wahlstrom T, Knuutila S and Butzow R (1998) Distinct chromosomal imbalances in uterine serous and endometrioid carcinomas. *Cancer Res* **58**: 892–895

Sonoda G, du Manoir S, Godwin AK, Bell DW, Liu Z, Hogan M, Yakushiji M and Testa JR (1997) Detection of DNA gains and losses in primary endometrial carcinomas by comparative genomic hybridization. *Genes Chromosomes Cancer* **18**: 115–125

### Gastroesophageal

Moskaluk CA, Hu J and Perlman EJ (1998) Comparative genomic hybridization of esophageal and gastroesophageal adenocarcinomas shows consensus areas of DNA gain and loss. *Genes Chromosomes Cancer* **22**: 305–311

### Gastrointestinal

El-Rifai W, Sarlomo-Rikala M, Miettinen M, Knuutila S and Andersson LC (1996) DNA copy number losses in chromosome 14: an early change in gastrointestinal stromal tumors. *Cancer Res* **56**: 3230–3233

### Gastric carcinoma

Koizumi Y, Tanaka S-I, Mou R, Koganei H, Kokawa A, Kitamura R, Yamauchi H, Ookubo K, Saito T, Tominaga S, Matsumura K, Shimada H, Tsuchida N and Sekihara H (1997) Changes in DNA copy number in primary gastric carcinomas by comparative genomic hybridization. *Clin Cancer Res* **3**: 1067–1076

Kokkola A, Monni O, Puolakkainen P, Larramendy ML, Victorzon M, Nordling S, Haapiainen R, Kivilaakso E and Knuutila S (1997) 17q12–21 amplicon, a novel recurrent genetic change in intestinal type of gastric carcinoma: a comparative genomic hybridization study. *Genes Chromosomes Cancer* **20**: 38–43

### Head and neck

Speicher MR, Howe C, Crotty P, du Manoir S, Costa J and Ward DC (1995) Comparative genomic hybridization detects novel deletions and amplifications in head and neck squamous cell carcinomas. *Cancer Res* **55**: 1010–1013

Weber RG, Scheer M, Born IA, Joos S, Cobbers JL, Hofele C, Reifenberger G, Zoller JE and Lichter P (1998) Recurrent chromosomal imbalances detected in biopsy material from oral premalignant and malignant lesions by combined tissue microdissection, universal DNA amplification, and comparative genomic hybridization. *Am J Pathol* **153**: 295–303

Wolff E, Girod S, Liehr T, Vorderwulbecke U, Ries J, Steininger H and Gebhart E (1998) Oral squamous cell carcinomas are characterized by a rather uniform pattern of genomic imbalances detected by comparative genomic hybridisation. *Oral Oncol* **34**: 186–190

### Liver

Marchio A, Meddeb M, Pineau P, Danglot G, Tiollais P, Bernheim A and Dejean A (1997) Recurrent chromosomal abnormalities in hepatocellular carcinoma detected by comparative genomic hybridization. *Genes Chromosomes Cancer* **18**: 59–65

### Lung

Balsara BR, Sonoda G, du Manoir S, Siegfried JM, Gabrielson E and Testa JR (1997) Comparative genomic hybridization analysis detects frequent, often high-level overrepresentation of DNA sequences at 3q, 5p, 7p and 8q in human non-small-cell lung carcinomas. *Cancer Res* **57**: 2116–2120

Björkqvist A-M, Tammilehto L, Anttila S, Mattson K and Knuutila S (1997) Recurrent DNA copy number changes in 1q, 4q, 6q, 9p, 13q, 14q and 22q detected by comparative genomic hybridization in malignant mesothelioma. *Br J Cancer* **75**: 523–527

© Cancer Research Campaign 1999

Björkqvist A-M, Tammilehto L, Nording S, Nurminen M, Anttila S, Mattson K and Knuutila S (1998*a*) Comparison of DNA copy number changes in malignant mesothelioma, adenocarcinoma and large-cell anaplastic carcinoma of the lung. *Br J Cancer* **77**: 260–269

Björkqvist A-M, Husgafvel-Pursiainen K, Anttila S, Karjalainen A, Tammilehto L, Mattson K, Vainio H and Knuutila S (1998*b*) DNA gains in 3q occur frequently in squamous cell carcinoma of the lung, but not in adenocarcinoma. *Genes Chromosomes Cancer* **22**: 79–82

Kivipensas P, Bjorkqvist A-M, Karhu R, Pelin K, Linnainmaa K, Tammilehto L, Mattson K, Kallioniemi O-P and Knuutila S (1996) Gains and losses of DNA sequences in malignant mesothelioma by comparative genomic hybridization. *Cancer Genet Cytogenet* **89**: 7–13

Petersen I, Langreck H, Wolf G, Schwendel A, Psille P, Vogt P, Reichel MB, Ried T and Dietel M (1997*a*) Small-cell lung cancer is characterized by a high incidence of deletions on chromosomes 3p, 4p, 5q, 10q, 13q and 17p. *Br J Cancer* **75**: 79–86

Petersen I, Bujard M, Petersen S, Wolf G, Goeze A, Schwendel A, Langreck H, Gellert K, Reichel M, Just K, duManoir S, Cremer T, Dietel M and Ried T (1997*b*) Patterns of chromosomal imbalances in adenocarcinoma and squamous cell carcinoma of the lung. *Cancer Res* **57**: 2331–2335

Ried T, Petersen I, Holtgreve-Grez H, Speicher MR, Schröck, du Manoir S and Cremer T (1994) Mapping of multiple DNA gains and losses in primary small-cell lung carcinomas by comparative genomic hybridization. *Cancer Res* **54**: 1801–1806

### Merkel cell carcinoma

Härle M, Arens N, Moll I, Back W, Schulz T and Scherthan H (1996) Comparative genomic hybridization (CGH) discloses chromosomal and subchromosomal copy number changes in Merkel cell carcinomas. *J Cutan Pathol* **23**: 391–397

### Neuroblastoma

Altura RA, Maris JM, Li H, Boyett JM, Brodeur GM and Look AT (1997) Novel regions of chromosomal loss in familial neuroblastoma by comparative genomic hybridization. *Genes Chromosomes Cancer* **19**: 176–184

Brinkschmidt C, Christiansen H, Terpe HJ, Simon R, Lampert F, Bocker W and Storkel S (1996) Synopsis of unbalanced chromosomal-aberrations in neuroblastoma by comparative genomic hybridization. *Pathologe* **17**: 368–373

Lastowska M, Cotterill S, Pearson AJ, Roberts P, McGuckin A, Lewis I and Bown N (1997) Gain of chromosome arm 17q predicts unfavourable outcome in neuroblastoma patients. *Eur J Cancer* **33**: 1627–1633

Lastowska M, Nacheva E, McGuckin A, Curtis A, Grace C, Pearson A and Brown N (1997) Comparative genomic hybridization study of primary neuroblastoma tumors. *Genes Chromosomes Cancer* **18**: 162–169

Plantaz D, Mohapatra G, Matthay KK, Pellarin M, Seeger RC and Feuerstein BG (1997) Gain of chromosome 17 is the most frequent abnormality detected in neuroblastoma by comparative genomic hybridization. *Am J Pathol* **150**: 81–89

Schütz BR, Scheurlen W, Krauss J, du Manoir S, Joos S, Bentz M and Lichter P (1996) Mapping of chromosomal gains and losses in primitive neuroectodermal tumors by comparative genomic hybridization. *Genes Chromosomes Cancer* **16**: 196–203

Szymas J, Wolf G, Kowalczyk D, Nowak S and Petersen I (1997) Olfactory neuroblastoma: detection of genomic imbalances by comparative genomic hybridization. *Acta Neurochir* **139**: 839–844

Van Gele M, Van Roy N, Jauch A, Laureys G, Benoit Y, Schelfhout V, De Porter CR, Brock P, Uyttebroeck A, Sciot R, Schuuring E, Versteeg R and Speleman F (1997) Sensitive and reliable detection of genomic imbalances in human neuroblastoma using comparative genomic hybridisation analysis. *Eur J Cancer* **33**: 1979–1982

### Neuroendocrine tumours of the digestive system

Terris B, Meddeb M, Marchio A, Danglot G, Flejou JF, Belghiti J, Ruszniewski P and Bernheim A (1998) Comparative genomic hybridization analysis of sporadic neuroendocrine tumors of the digestive system. *Genes Chromosomes Cancer* **22**: 50–56

### Ovary

Arnold N, Hägele L, Walz L, Schempp W, Pfisterer J, Bauknecht T and Kiechle M (1996) Over-represenation of 3q and 8q material and loss of 18q material are recurrent findings in advanced human ovarian cancer. *Genes Chromosomes Cancer* **16**: 46–54

Iwabuchi H, Sakamoto M, Sakunaga H, Ma Y-Y, Carcangiu ML, Pinkel D, Yang-Feng TL and Gray JW (1995) Genetic analysis of benign, low-grade, and high-grade ovarian tumors. *Cancer Res* **55**: 6172–6180

Riopel MA, Spellerberg A, Griffin CA and Perlman EJ (1998) Genetic analysis of ovarian germ cell tumors by comparative genomic hybridization. *Cancer Res* **58**: 3105–3110

Tapper J, Sarantaus L, Vahteristo P, Nevanlinna H, Hemmer S, Seppala M, Knuutila S and Butzow R (1998) Genetic changes in inherited and sporadic ovarian carcinomas by comparative genomic hybridization: extensive similarity except for a difference at chromosome 2q24–q32. *Cancer Res* **58**: 2715–2719

Sonoda G, Palazzo J, du Manoir S, Godwin AK, Feder M, Yahushiji M and Testa JR (1997) Comparative genomic hybridisation detects frequent overrepresentation of chromosomal material from 3q26, 8q24 and 20q13 in human ovarian carcinomas. *Genes Chromosomes Cancer* **20**: 320–328

Tapper J, Bützow R, Wahlstöm T, Seppälä M and Knuutila S (1997) Evidence for divergence of DNA copy number changes in serous, mucinous and endometrioid ovarian carcinomas. *Br J Cancer* **75**: 1782–1787

### Pancreas

Fukushige S, Waldman FM, Kimura M, Abe T, Furukawa T, Sunamura M, Kobari M and Horii A (1997) Frequent gain of copy number on the long arm of chromosome 20 in human pancreatic adenocarcinoma. *Genes Chromosomes Cancer* **19**: 161–169

Mahlamäki EH, Höglund M, Gorunova L, Karhu R, Dawiskiba S, Andrén-Sandberg Å, Kallionemi OP and Johansson B (1997) Comparative genomic hybridisation reveals frequent gains of 20q, 8q, 11q, 12p and 17q, and losses of 18q, 9p and 15q in pancreatic cancer. *Genes Chromosomes Cancer* **20**: 383–391

Solinas-Toldo S, Wallrapp C, Müller-Pillasch F, Bentz M, Gress T and Lichter P (1996) Mapping of chromosomal imbalances in pancreatic carcinoma by comparative genomic hybridization. *Cancer Res* **56**: 3803–3807

### Parathyroid

Palanisamy N, Imanishi Y, Rao PH, Tahara H, Chaganti RK and Arnold A (1998) Novel chromosomal abnormalities identified by comparative genomic hybridization in parathyroid adenomas. *J Clin Endocr Metab* **83**: 1766–1770

### Pituitary

Daniely M, Aviram A, Adams EF, Buchfelder M, Barkai G, Fahlbusch R, Goldman B and Friedman E (1998) Comparative genomic hybridization analysis of nonfunctioning pituitary tumors. *J Clin Endocr Metab* **83**: 1801–1805

### Prostate

Cher ML, MacGrogan D, Bookstein R, Brown JA, Jenkins RB and Jensen RH (1994) Comparative genomic hybridization, allelic imbalance, and fluorescence in situ hybridization on chromosome 8 in prostate cancer. *Genes Chromosomes Cancer* **11**: 153–162

Nupponen NN, Kakkola L, Koivisto P and Visakorpi T (1998) Genetic alterations in hormone-refractory recurrent prostate carcinomas. *Am J Pathol* **153**: 141–148

Joos S, Bergerheim USR, Pan Y, Matsuyama H, Bentz M, du Manoir S and Lichter P (1995) Mapping of chromosomal gains and losses in prostate cancer by comparative genomic hybridization. *Genes Chromosones Cancer* **14**: 267–276

Visakorpi T, Kallioniemi AH, Syvänen A-C, Hyytinen ER, Karhy R, Tammela T, Isola J and Kallioniemi O-P (1995) Genetic changes in primary and recurrent prostate cancer by comparative genomic hybridization. *Cancer Res* **55**: 342–347

### Renal

Bentz M, Bergerheim UR, Li C, Joos S, Werner CA, Baudis M, Gnarra J, Merino MJ, Zbar B, Linehan WM and Lichter P (1996) Chromosome imbalances in papillary renal cell carcinoma and first cytogenetic data of familial cases analyzed by comparative genomic hybridization. *Cyto Cell Genet* **75**: 17–21

Gronwald J, Storkel S, Holtgreve Grez H, Hadaczek P, Brinkschmidt C, Jauch A, Lubinski J and Cremer T (1997) Comparison of DNA gains and losses in primary renal clear cell carcinomas and metastatic sites: importance of 1q and 3p copy number changes in metastatic events. *Cancer Res* **57**: 481–487

 © Cancer Research Campaign 1999

Moch H, Presti JC, Sauter G, Buchholz N, Jordan P, Mihatsch MJ and Waldman FM (1996) Genetic aberrations detected by comparative genomic hybridization are associated with clinical outcome in renal cell carcinoma. *Cancer Res* **56**: 27–30

Presti JC, Moch H, Reuter VE, Huynh D and Waldman FM (1996) Comparative genomic hybridization for genetic analysis of renal oncocytomas. *Genes Chromosomes Cancer* **17**: 199–204

Presti JC, Moch H, Reuter VE, Cordoncardo C and Waldman FM (1996) Renal-cell carcinoma genetic-analysis by comparative genomic hybridization and restriction fragment length polymorphism analysis. *J Urol* **156**: 281–285

### Rhabdomyosarcoma

Weber-Hall S, Anderson J, McManus A, Abe S, Nojima T, Pinkerton R, Pritchard-Jones K and Shipley J (1996) Gains, losses, and amplification of genomic material in rhabdomyosarcoma analyzed by comparative genomic hybridization. *Cancer Res* **56**: 3320–3224

### Sarcoma

Armengol G, Tarkkanen M, Virolainen M, Forus A, Valle J, Bohling T, AskoSeljavaara S, Blomqvist C, Elomaa I, Karaharju E, Kivioja AH, Siimes MA, Tukiainen E, Caballin MR, Myklebost O and Knuutila S (1997) Recurrent gains of 1q, 8 and 12 in the Ewing family of tumours by comparative genomic hybridization. *Br J Cancer* **75**: 1403–1409

Forus A, Olde Weghuis D, Smeets D, Fodstad Ø, Myklebost O and van Kessel A-G (1995) Comparative genomic hybridization analysis of human sarcomas: 1. Occurrence of genomic imbalances and identification of a novel major amplicon at 1q21-q22 in soft tissue sarcomas. *Genes Chromosomes Cancer* **14**: 8–14

Forus A, Olde Weghuis D, Smeets D, Fodstad Ø, Myklebost O and van Kessel AG (1995) Comparative genomic hybridization analysis of human sarcomas: II. Identification of novel amplicons at 6p and 17p in osteosarcomas. *Genes Chromosomes Cancer* **14**: 15–21

KiuruKuhlefelt S, ElRifai W, SarlomoRikala M, Knuutila S and Miettinen M (1998) DNA copy number changes in alveolar soft part sarcoma: a comparative genomic hybridization study. *Mod Path* **11**: 227–231

Larramendy ML, Tarkkanen M, Blomqvist C, Virolainen M, Wiklund T, AskoSelijavaara S, Elomaa I and Knuutila S (1997*a*) Comparative genomic hybridization of malignant fibrous histiocytoma reveals a novel prognostic marker. *Am J Pathol* **151**: 1153–1161

Larramendy ML, Valle J, Tarkkanen M, Kivioja AH, Karaharju E, Salmivalli T, Elomma I and Knuutila S (1997*b*) No DNA copy number changes in osteochondromas: a comparative genomic hybridization study. *Cancer Genet Cytogenet* **97**: 76–78

Packenham JP, duManoir S, Schrock E, Risinger JI, Dixon D, Denz DN, Evans JC, Berchuck A, Barrett JC, Devereux TR and Ried T (1997) Analysis of genetic alterations in uterine leiomyomas and leiomyosarcomas by comparative genomic hybridization. *Mol Carcinogen* **19**: 273–279

Szymanska J, Tarkkanen M, Wiklund T, Virolainen M, Blomqvist C, Asko-Seljavaara S, Tukiainen E, Elomaa I and Knuutila S (1996) Gains and losses of DNA sequences in liposarcomas evaluated by comparative genomic hybridization. *Genes Chromosomes Cancer* **15**: 89–94

Szymanska J, Mandahl N, Mertens F, Tarkkanen M, Karaharju E and Knuutila S (1996) Ring chromosomes in parosteal osteosarcoma contain sequences from 12q13–15: a combined cytogenetic and comparative genomic hybridization study. *Genes Chromosomes Cancer* **16**: 31–34

### Testicular

Decker H-JH, Neuhaus C, Jauch A, Speicher M, Ried T, Bujard M, Brauch H, Störkel S, Stöckle M, Selger B and Huber C (1996) Detection of a germline mutation and somatic homozygous loss of the von Hippel-Lindau tumor-suppressor gene in a family with a de novo mutation. A combined genetic study, including cytogenetics, PCR/SSCP, FISH, and CGH. *Hum Genet* **97**: 770–776

Korn WM, Olde Weghuis DEM, Suijkerbuijk RF, Schmidt U, Otto T, du Manoir S, van Kessel AG, Harstrick A, Seeber S and Bechre R (1996) Detection of chromosomal DNA gains and losses in testicular germ cell tumours by comparative genomic hybridization. *Genes Chromosomes Cancer* **17**: 78–87

Mostert MMC, van de Pol M, Olde Weighuis D, Suijkerbuijk RF, van Kessel AG, van Echten J, Oosterhuis JW and Looijenga LHJ (1996) Comparative genomic hybridization of germ cell tumors of the adult testis: confirmation of karyotypic findings and identification of a 12p-amplicon. *Cancer Genet Cytogenet* **89**: 146–152

Ottesen AM, Kirchhoff M, Rajpert EW, Maahr J, Gerdes T, Rose H, Lundsteen C, Petersen PM, Philip J and Skakkebaek NE (1997) Detection of chromosomal aberrations in seminomatous germ cell tumours using comparative genomic hybridisation. *Genes Chromosomes Cancer* **20**: 412–418

Speicher MR, Jauch A, Walt H, du Manoir S, Ried T, Jochum W, Sulser T and Cremer T (1995) Correlation of microscopic phenotype with genotype in a formalin-fixed, paraffin-embedded testicular germ cell tumor with universal DNA amplification, comparative genomic hybridization, and interphase cytogenetics. *Am J Pathol* **146**: 1332–1340

### Uveal melanoma

Ghazvini S, Char DH, Kroll S, Waldman FM and Pinkel D (1996) Comparative genomic hybridization analysis of archival formalin-fixed paraffin-embedded uveal melanomas. *Cancer Genet Cytogenet* **90**: 95–101

Speicher MR, Prescher G, du Manoir S, Jauch A, Horsthemke B, Bornfeld R, Becher R and Cremer T (1994) Chromosomal gains and losses in uveal melanomas detected by comparative genomic hybridization. *Cancer Res* **54**: 3817–3838

### Wilms' tumour

Steenman M, Redeker B, deMeulemeester M, Wiesmeijer K, Voute PA, Westerveld A, Slater R and Mannens M (1997) Comparative genomic hybridization analysis of Wilms' tumors. *Cytogenet Cell Genet* **77**: 296–303

Valentine RAAM, Li H, Boyett JM, Shearer P, Grundy P, Shapiro DN and Look T (1996) Identification of novel regions of deletion in familial Wilms' tumor by comparative genomic hybridization. *Cancer Res* **56**: 3837–3841

*© Cancer Research Campaign 1999*
